# Tracheobronchial calcifications in a child

**DOI:** 10.11604/pamj.2020.36.331.25384

**Published:** 2020-08-24

**Authors:** Houda Rahmoun, Chafiq Mahraoui

**Affiliations:** 1 Department of Pediatrics I, Children´s Hospital of Rabat, Rabat, Morocco

**Keywords:** Tracheobronchial calcifications, Keutel syndrome, child

## Image in medicine

Three months old child was admitted in the department of pediatrics I for respiratory distress, fever and laryngeal stridor. He was born to consanguineous parents. The prenatal history was unremarkable. However, he was hospitalized at birth for respiratory distress which improved quickly with oxygen nasal cannula. Since one month old, he was presenting a laryngeal stridor, persistent cough and chest congestion. Clinical examination found a dyspnea, stridor occuring in both phases of respiration, signs of retractions, a dysmorphic face with mid-facial hypoplasia and brachytelephalengia. The chest x-ray showed calcifications involving the entire tracheobronchial tree. A chest computed tomography revealed bilateral and symmetrical calcifications involving the anterior cartilaginous part of the trachea and the stem bronchi. The transthoracic ultrasound was normal. Routine physicochemical examinations found a low prothrombin time with decrease in the levels of vitamin-K dependent coagulation factors. The clinical course was favorable with oxygen nasal cannula and respiratory physiotherapy. The diagnosis of Keutel syndrome was made on calcifications of the tracheal cartilage associated to brachytelephalengia, and also facial dysmorphism.

**Figure 1:**
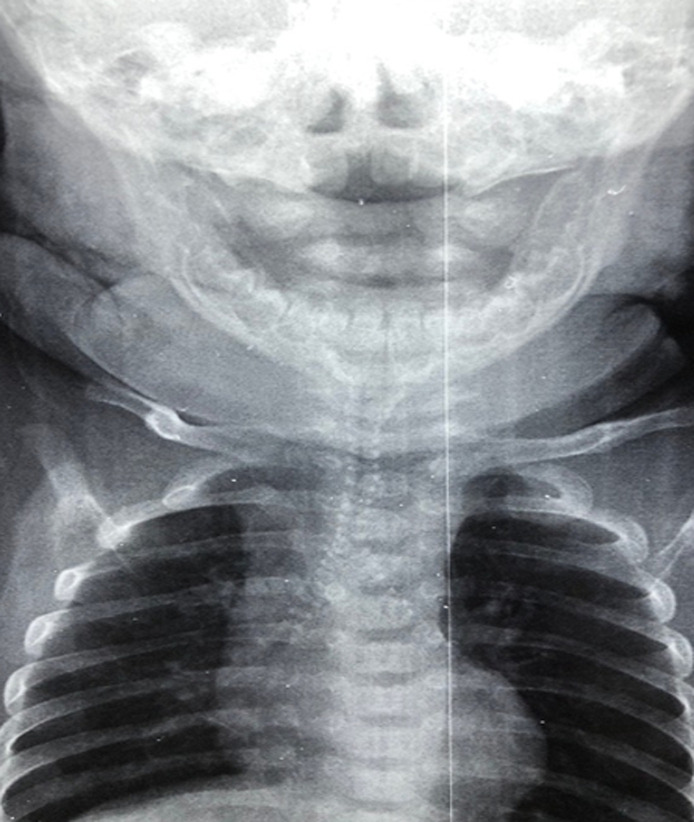
tracheobronchial calcifications

